# Olfactory Dysfunction, Headache, and Mental Clouding in Adults with Long-COVID-19: What Is the Link between Cognition and Olfaction? A Cross-Sectional Study

**DOI:** 10.3390/brainsci12020154

**Published:** 2022-01-24

**Authors:** Arianna Di Stadio, Michael J. Brenner, Pietro De Luca, Maria Albanese, Luca D’Ascanio, Massimo Ralli, Dalila Roccamatisi, Cristina Cingolani, Federica Vitelli, Angelo Camaioni, Stefano Di Girolamo, Evanthia Bernitsas

**Affiliations:** 1 Department GF Ingrassia, University of Catania, 95123 Catania, Italy; 2 Department of Otolaryngology-Head and Neck Surgery, University of Michigan Medical School, Ann Arbor, MI 48105, USA; mbren@med.umich.edu; 3 Department of Medicine, Surgery and Dentistry, University of Salerno, 84125 Salerno, Italy; dr.dlp@hotmail.it; 4 Department of Systems Medicine, University of Rome “Tor Vergata”, 00133 Rome, Italy; maria.albanese@hotmail.it; 5 Department of Otolaryngology, AORMN, 61032 Fano, Italy; l.dascanio@gmail.com (L.D.); cristina.cingolani@ospedalimarchenord.it (C.C.); fe.vitelli@gmail.com (F.V.); 6 Organ of Sense Department, University La Sapienza, 00185 Rome, Italy; massimo.ralli@uniroma1.it; 7 Psychology Department, UTIU, 00133 Rome, Italy; dalilaviolet@hotmail.com; 8 Otolaryngology Department, San Giovanni Addolorata Hospital, 00133 Rome, Italy; acamaioni@hsangiovanni.roma.it; 9 Unit of Otorhinolaryngology, Department of Clinical Sciences and Translation Medicine, Tor Vergata University, 00133 Rome, Italy; Stefano.digirolamo@uniroma2.it; 10 Multiple Sclerosis Center, Neurology Department, Wayne State University, Detroit, MI 48201, USA; ebernits@med.wayne.edu

**Keywords:** smell, brain fog, mental clouding, headache, COVID-19, SARS-CoV-2, neuroinflammation, olfaction, olfactory dysfunction, cognitive deficit

## Abstract

Smell alteration and cognitive impairment are common features of the Long-COVID Syndrome. Mental clouding, often described as brain fog, might affect smell by altering recollection of odors or through a share mechanism of neuroinflammation. We investigated mental clouding, headache, and cognitive function in adult patients with persistent COVID-19 olfactory dysfunction. This multi-center cross-sectional study enrolled 152 adults with self-reported olfactory dysfunction from 3 tertiary centers specialized in COVID-19 olfactory disorders. Inclusion criteria were smell alterations after COVID-19 persisting over 6 months from infection, age >18 and < 65. Exclusion criteria included smell alterations, headache, or memory problems prior to COVID-19 infection. The patients were evaluated by olfactometry, nasal endoscopy, headache scale, cognitive assessment, Mini Mental State Examination (MMSE), and self-reported measures. Smell dysfunction was stratified and classified based on olfactory deficit severity and presence of olfactory distortion (parosmia, cacosmia). Data on smell disorder, mental clouding, MMSE, and headache were analyzed to assess correlations. Among the 152 patients studied, 50 (32.8%) presented with anosmia, 25 (16.4%) with hyposmia, 10 (6.6%) with parosmia/cacosmia, and 58 patients (38.2%) with a combination of hyposmia and parosmia; seven (4.6%) patients suffered from headache exclusively, and two (1.4%) had headache and mental clouding as their primary symptom. Headache was reported by 76 (50%) patients, and mental clouding by 71 (46.7%). The patients reporting headache, mental clouding, or both, had significantly increased risk of suffering from anosmia and/or hyposmia when compared with their counterparts without these neurological symptoms. No patients had reduced MMSE scores. In our cohort of adult patients with post-COVID-19, smell alterations persisting over 6 months, cognitive impairment and headache were associated with more severe olfactory loss, consistent with neuroinflammatory mechanisms mediating a variety of Long-COVID symptoms.

## 1. Introduction

COVID-19 has affected more than 275,000,000 people worldwide, with a wide range in clinical presentation and duration of symptoms. Initially considered primarily a respiratory illness, Severe Acute Respiratory Syndrome Coronavirus 2 (SARS-CoV-2) is now recognized as a multi-organ disease. The infection, spreading from the nasopharynx into the brain [1,2], causes anosmia [3] and several neurologic symptoms [4,5], ranging from impaired executive function to hearing and vestibular dysfunction [1]. The respiratory infection and, when present, the olfactory and cognitive impairment tend to spontaneously dissipate within weeks following the infection [3,4], especially in patients with mild to moderate disease.

Persistence of neurologic symptoms is more common than initially recognized [1,4,5]. The propensity of SARS-CoV-2 to cause post-acute symptoms, such as fatigue, headache, altered memory/thinking, and joint pain or muscle aches, has led to coining of the term “Long-COVID Syndrome”, when symptoms persist over 6 months after swab negativization [6]. Additionally referred to as long-haul COVID, or post-acute COVID-19 [7], the entity remains poorly understood and much work remains in refining the diagnosis, etiology, associations, and management of this evolving concept, despite several authors’ having tried to better define the condition [8,9,10].

Fatigue and persistent difficulty with memory, concentration, and decision-making are among the most frequent neurological symptoms in non-hospitalized ‘long-hauler’ patients with COVID-19; furthermore, many patients report subtle cognitive impairment and behavioral changes that may be difficult for them to characterize. These symptoms are often collectively referred to as “brain fog” [11]; we use the term “mental clouding” to describe this phenomenon often reported by the patients. 

Mental clouding has been attributed to either selective neuronal mitochondrial dysfunction [11] or neuroinflammatory events, which arise following spread of the virus into the memory centers. The spread of the inflammation from the periphery to the olfactory bulbs and higher brain centers, has been observed in hearing and vestibular disorders [1]. Because cognitive and olfactory functions are interdependent [12], investigating the correlation between these entities could provide insights into a common pathway for the deficits that characterize Long-COVID Syndrome. We, therefore, analyzed the prevalence of mental clouding, memory deficit, and headache in patients with COVID-19 chronic olfactory dysfunction and then calculated Odds Ratio comparing the patients who presented only olfactory disorders with the ones suffering from smell disorders and neurological symptoms. 

## 2. Materials and Methods

This study included 152 consecutive outbound adult patients (102 females and 50 males, average age 41.2 ± 11) who presented to any of the three COVID-19 Smell Disorder Centers (in Italy) with self-reported smell disorders related to COVID-19. Patients were recruited from May to September 2021. The study was coordinated by one PI (ADS), and all centers collected data about the patients in an identical manner, using the same instruments. 

All patients reported persistent olfactory alteration after SARS-CoV-2 infection; the diagnosis of COVID-19 was confirmed by reverse transcription-polymerase chain reaction nasopharyngeal swab. The study was approved by the hospital IRB, and all participants signed a written consent in which they approved the use of their data for scientific study. The study was performed in accordance with the Declaration of Helsinki ethical standards. 

The following exclusion and inclusion criteria were applied:

The inclusion criteria were: smell alteration after COVID-19 infection persistent over 6 months after swab negativization, 18–65 years of age, consent to participate, pre-COVID normal cognitive function, and absence of severe visual or hearing deficits.

The exclusion criteria were: presence of chronic smell alteration, headache, or memory problems prior to onset of COVID-19 infection. We also excluded patients with history of any of the following: stroke, neurological/psychiatric disorders, including Alzheimer and Parkinson diseases, and cancer requiring either radiation or chemotherapy within three years from the time of recruitment. Active smokers were also excluded.

We collected data on gender, age, comorbidities, smoking history, presence of headache, and, if applicable, headache characteristics (area and intensity). We also recorded the length of symptoms and persistence of symptoms after resolution of acute COVID-19.

### 2.1. Headache Evaluation

We used the Wong-Baker FACES Pain Rating Scale to measure headache severity [13]. This assessment scores pain using the following anchors: 0 is no pain, 1 to 2 refers to mild pain, 3 to 4 refers to moderate pain, 5–6 refers to severe pain, 7–8 refers to very severe pain, and 9–10 is worst pain possible.

### 2.2. Memory Investigation

Cognitive function was assessed using the Mini Mental State Examination (MMSE) test and clinical questions.

To test for mental clouding, we asked the following questions: (i) Do you forget information that you recently learned, resulting in difficulty performing a task?; (ii) Do you have to ask for information to be repeated or have an increased need for reminder notes?; (iii) Have you experienced difficulty in remembering common names of objects or persons?; and (iv) Has your concentration, memory, or overall mental ability deteriorated?

Three answers were possible: (A) I suffered this problem temporarily, but it resolved (no residual memory/cognitive impairment); (B) I am suffering from this problem currently (memory/cognitive alteration is still persistent); and (C) I have never experienced this problem (absence of cognitive impairment related to COVID-19).

Patients were classified as suffering from mental clouding if they answered, “I am still suffering from this problem currently” for any of the 4 areas queried; this positive answer (B) had to be selected for all four question to classify patients as affected by mental clouding.

### 2.3. Nasal Endoscopy and Olfactory Testing

All patients underwent nasal endoscopy to exclude nasal conditions that could negatively affect the olfactory testing. If masses, sinonasal polyposis, active infection, or other rhinological disorders were identified on nasal endoscopy, the patients were excluded from the study. Patients were also queried regarding history of prior impaired smell, history of nasal/ nasopharyngeal malignancy, history of radiation, or other anatomical abnormalities that would interfere with sense of smell.

To evaluate olfactory function, Burghart Sniffin’ Sticks identification test (16 pen test) was performed, as directed by the manufacturer (MediSense, Sense Trading BV, Groningen—Netherlands). After performing the test, the correct answers were counted, and the responses were recorded on the evaluation pad Sniffin’ stick. Based on the score, the patient was classified: 13–16: normosmia, 12–8: hyposmia, and 0–7: anosmia Additionally, all patients were queried regarding the perception of altered olfaction (parosmia) and about aversive smell (cacosmia).

### 2.4. Statistical Analysis

The prevalence of smell disorders, cognitive decline, and headache was determined. We calculated Odds Ratio (OR) looking at the single neurological symptom (mental clouding or headache) or the combination of them related the different smell disorders (anosmia, hyposmia, parosmia, hyposmia + parosmia); due to the nature of the test used (OR), the smell disorders were used as nominal instead of numeric data, so we used the clinical classification of the symptoms instead of the numeric values obtained by the Sniffin’ test. The statistical value was considered significant at *p* < 0.05. Statistical tests were performed by Stata^®^.

## 3. Results

Among the 152 participants, none had been hospitalized for SARS-CoV-2 infection. Persistent smell alteration was present for a mean period of 9.8 + 2.8 months after a negative swab for SARS-CoV-2.

None of the patients had visible evidence of nasal mucosa inflammation at the time of the screening.

**Figure 1:**
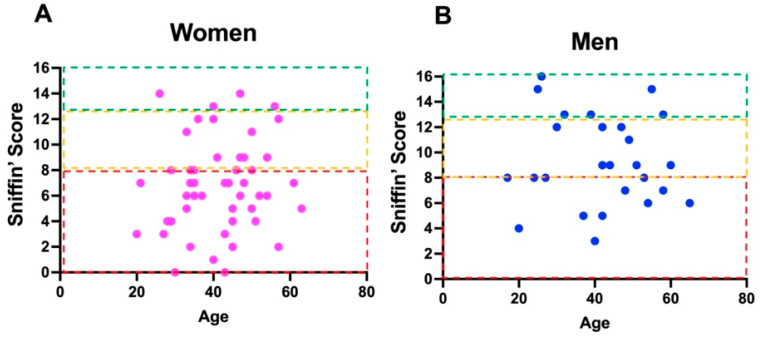
(**A**) Distribution for age of different Sniffin’ scores in women. (**B**) Distribution for age of different Sniffin’ scores in men. The boxes in red, yellow, and green color represent the normal range for general population in term of normality, hypofunction or absence of smell capacity. Dots in the red boxes are indicative of anosmia, in the yellow are of hyposmia, and in the green are of normosmia. The reference value for normal olfactory scores refers to the data published by Oleszkiewicz et al. [14].

**Figure 2:**
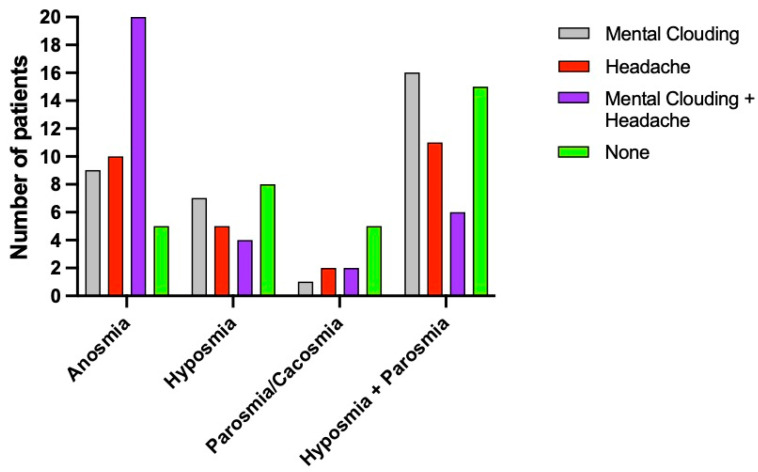
Distribution of neurological symptoms and smell alterations.

50 patients (32.8 %) presented with anosmia, 25 (16.4%) with hyposmia (Figure 1), 10 (6.6%) with parosmia/cacosmia, 58 (38.2%) with a combination of hyposmia and parosmia, seven (4.6%) patients suffered from headache exclusively, and two (1.4%) had only headache and mental clouding as their primary symptom (Figure 2).

All patients were healthy with no significant comorbidities; none of the patients were affected by major systemic diseases, such as cardiovascular disorders, diabetes, or thyroid dysfunction.

Considering all patients (with and without olfactory alteration) and the neurological symptom (headache or mental clouding or coexistence of both), 76 patients reported headache (50%) and 71 mental clouding (46.7%).

No patients suffered from memory problems prior to COVID-19 infection. MMSE performed at time of study were in the normal range for all patients. Within the cohort of patients with olfactory disorders (143/152), 34 (23.7%) reported mental clouding based on the self-report questionnaire; 34 patients (23.8%) reported headache; and 35 patients (24.5%) reported both headache and mental clouding. 40 patients (28%) had no neurological symptoms associated to their smell alterations. Most patients with headache (single or associate with mental clouding) (62.6%, or 42 people) reported frontal headache, with the remaining patients describing a diffuse headache. None of the patients described their headache as severe or worst possible headache. Considering both patients with headache only and the ones who had headache associated with mental clouding (67 people), 85% patients (57 individuals) reported mild headache; and the remainder reported moderate headache (10 patients, 25%).

**Figure 3:**
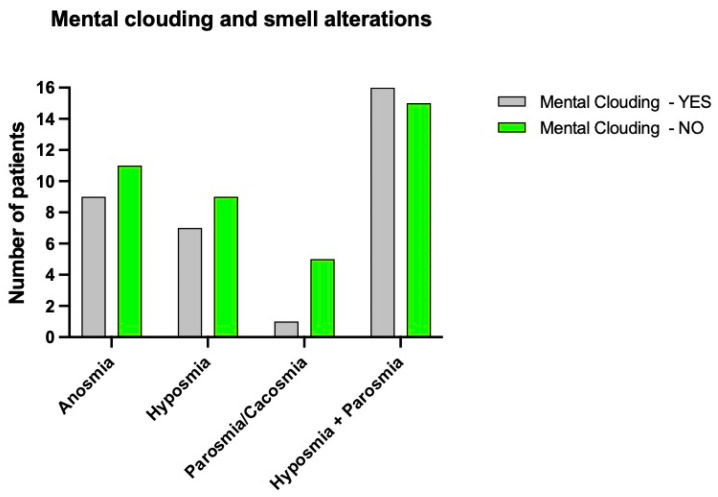
Distribution of mental clouding (brain fog) (presence and absence) relative to the different smell alterations.

Mental clouding alone was found in 9 subjects (18%) with anosmia, in 7 subjects (28%) with hyposmia, in 1 individual (10%) with parosmia, and in 16 (27.5%) who suffered from hyposmia and parosmia. Figure 3 shows the symptoms distribution related to the presence or absence of mental clouding.

**Figure 4:**
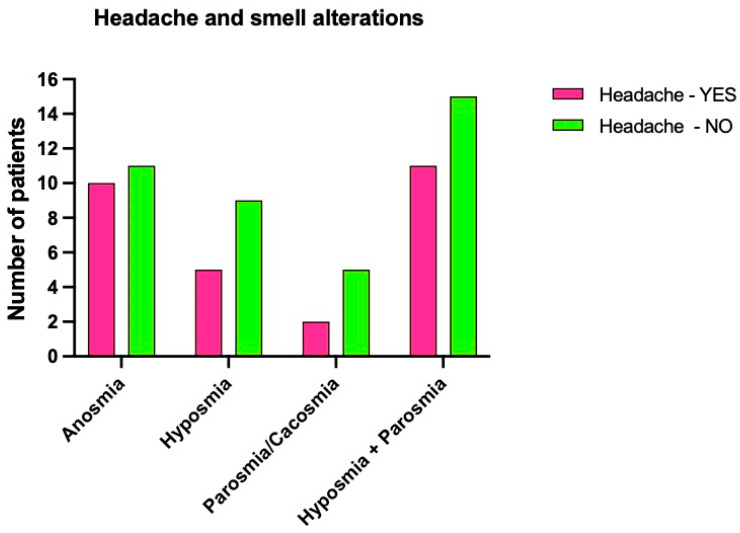
Shows smell alterations patients that reported headache.

Headache was present in 20% of people (10 persons) with anosmia, 20% (5 patients) with hyposmia, 20% (2 people) with parosmia, and in 11 (18.9%) who suffered from hyposmia and parosmia (Figure 4).

**Figure 5:**
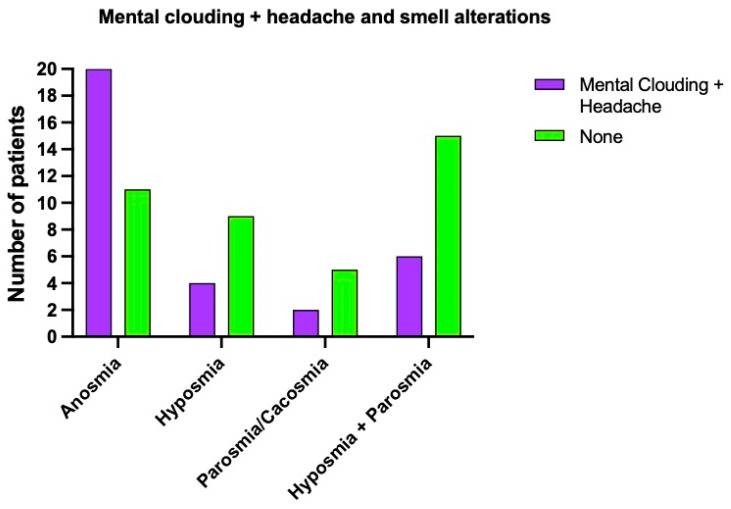
Smell alterations patients who reported both mental clouding and headache.

The combination of mental clouding and headache was present in 40% of our cohort (20 persons) with anosmia, 16% (4 patients) with hyposmia, 20% (2 patients) with parosmia, and in 10.3% (6 patients) who suffered from hyposmia and parosmia. Among patients who did not experience neurological symptoms, 22% (11 individuals) had anosmia, 36% (9 individuals) had hyposmia, 50% (5 individuals) suffered from parosmia, and 25.8% (15 individuals) with hyposmia and parosmia (Figure 5). There was no statistically significant difference between women and men (*p* = 0.8); 24.5% women and 30.6% men had neither headache nor mental clouding.

Patients with mental clouding had higher risk of suffering from anosmia (OR: 19; *p* = 0.05) or hyposmia + parosmia (OR: 33; *p* = 0.01), hyposmia alone (OR: 15; *p* = 0.07), and moderate risk of suffering from parosmia (OR: 3; *p* = 0.5) compared to patients with no neurological symptoms. Statistically significant *p* values were identified for anosmia (0.05) and hyposmia + parosmia (0.01), while hyposmia and parosmia were not statistically significant *p* values, respectively, 0.07 and 0.5.

In the presence of headache, the patients had increased risk of anosmia (OR: 21; *p* = 0.04) or hyposmia + parosmia (OR: 23; *p* = 0.03) and hyposmia (OR: 11; *p* = 0.12), and moderate risk of developing parosmia (OR: 5; *p* = 0.3), compared to patients with no neurological symptoms (Figure 2). Statistically significant *p* values were identified for anosmia (0.04) and hyposmia+ parosmia (0.03), while hyposmia and parosmia were not statistically significant *p* values, respectively, 0.12 and 0.3.

Risk comparison between individuals with mental clouding versus headache for development of smell abnormalities showed mental clouding sufferers had an increased risk of developing hyposmia + parosmia (OR: 33; *p* = 0.01) or parosmia (OR: 28.2; *p* = 0.05) and increased risk for anosmia (OR: 19; *p* = 0.05) or hyposmia (OR: 15; *p* = 0.08). Statistically significant *p* values were identified for hyposmia+ parosmia (0.01), while parosmia, anosmia and hyposmia were not statistically significant *p* values, respectively, 0.05, 0.05, and 0.08

Patients who were affected by both mental clouding and headache showed increased risk of presenting with anosmia (OR: 41; *p* = 0.01), hyposmia + parosmia (OR: 13; *p* = 0.08), or hyposmia alone (OR: 9; *p* = 0.1), and moderate risk of parosmia (OR: 5; *p* = 0.3).

Statistically significant *p* values were identified for anosmia (0.01) only. The other values were all not statistically significant hyposmia+ parosmia (0.08), hyposmia (0.1), and parosmia (0.3).

## 4. Discussion

In our cohort of Long-COVID patients with olfactory impairment, we identified a high prevalence of headache and mental clouding. More than half of the participants (60%) had suffered from persistent anosmia for >6 months (mean duration 9.8 ± 2.8 months) after COVID-19 resolution. The demographic characteristics of our study confirm previous observations of a higher rate of olfactory disorders and Long-COVID-19 in women [15]. Smell alterations are attributed to neuroinflammation of the olfactory bulb [16] and women, perhaps more predisposed to (neuro)inflammation, already have a higher baseline rate of olfactory bulbs atrophy with aging compared with men in the same age range [17]. We speculate that SARS-CoV-2 infection may ulterior increase this risk [18] because of neuroinflammation; similarly the infection might act on other neuroinflammatory and neurodegenerative diseases [19], including Alzheimer Disease (AD) and Parkinson Disease [19]. Because AD is more prevalent in women than men, [20] and SARS-CoV-2 inflammation can spread from the olfactory bulbs to other parts of the brain [21] increasing the neuroinflammation rate, we hypothize that anosmia may portend future neuro-degenerative neurological disease in predisposed subjects [18,22], as for example women. 

Interestingly, we found no correlation between mental clouding and MMSE score. We speculate that this lack of correlation between the presence of mental clouding and MMSE results could be related (i) to young patients’ age, in which cognitive impairment is uncommon and unlikely to be detected by MMSE; and (ii) small alterations in the memory abilities (minimal inflamed areas in the mnemonic pathways) which cause only small deficit not detectable by MMSE due to the poor sensitivity of the test.

Submental clouding might have diffused, nonspecific effects on neurocognitive function that fall below the threshold of detection of the MMSE, which has limited sensitivity.

We also speculate that the low incidence of memory difficulties in MMSE in our cohort may reflect selection bias, and such results may not necessarily apply to inpatient or other institutional care settings.

Despite the negative findings on MMSE, cognitive, physical, and psychological factors all contribute to the burden of COVID-19 survivorship [23].

Olfactory testing outcomes can also be influenced by patients’ adherence and attentiveness during the test, so mental clouding might interfere with odor detection and discrimination, explaining the increased risk we observed in patients with persistent anosmia and hyposmia + parosmia, compared to patients who did not suffer from memory alterations.

Mental clouding, as well as anosmia, hyposmia, and parosmia, might be manifestations of diffuse neuro-inflammation [21,24,25]. Song et al. reported that viral neurotropism may cause neuroinflammation in the brain and could account for systematic symptoms, such as cough, in patients with a normal upper and lower respiratory tract [26]. Neuroinflammation may underpin post-COVID cognitive impairment [25,27]. De Malo et al. showed that the olfactory bulb is a conduit for viral entry into the brain [21,27]; thus, persistence of the virus in the olfactory bulb could explain the persistence of smell alteration. Other data are conflicting; Khan et al. found that virus can affect the neuroepithelium without spreading into the olfactory bulbs [28]; however, the authors did not mention whether the patients suffered (or not) of smell disorders.

Viral neurotropism has not been confirmed yet, but we currently know that the virus can cause diffuse inflammation responsible for different neurological symptoms. From an anatomical point of view, the inflammation in the olfactory bulbs might spread in the frontal lobe -prefrontal cortex [29] and create a delay in the memory processing perceived by the patients as mental clouding [30,31].

There has been previous speculation that olfactory loss may be a harbinger of neurodegeneration, mediated by neuroinflammation, as proposed by Doty [32,33]. Our results align with the findings of Song [26], De Malo [27], and Xydakis et al., which suggest that persistence of smell disorders might increase the likelihood of developing long term-neurological disease [34]. We hypothesize that mental clouding arising from neuroinflammation could also affect the capacity of identifying the odors due to the memory disturbance (remembering and odor, or its name) with consequent low scores on the TDI Sniffin’ test [35]. Although mental clouding (“brain fog”) has been described as one of the symptoms of Long-COVID [36], the researchers identified a higher risk of developing this symptom in patients with parosmia only [37]. Our study is the first to identify an increased risk of anosmia and hyposmia+ parosmia in the patients suffering from mental clouding; we believe that these observations might help to explain the associations between these symptoms.

We identified 61.8% of patients with headache; these individuals carried a higher risk of suffering from anosmia and hyposmia with parosmia compared to the patients with only olfactory symptoms. In a previous study [3], we already observed the co-presence of headache and smell disorders in the COVID-19 population, but the prevalence of this symptom was slightly lower than the one observed in the present study (52% versus 61.8%). Our patients typically described their headache as frontal, transitory, and intense, arising 24–48 h before the smell alteration. Due to these characteristics, we attribute the headache to different conditions arising after SARS-CoV-2 viral entry; after entry via the nasopharynx, it may irritate the trigeminal nerve with consequent headache inflaming the olfactory bulb [3].

In this study, patients with headache suffered from severe and persistent smell disorders—we speculate [9], because the nasal mucosa was normal at the endoscopic investigation that it might be a sign of ongoing olfactory bulb inflammation [1,21,25,27]; however, due to inability of endoscopic investigation to detect micro-alteration of the mucosa at cellular level and the absence of MRI and biopsy of nasal bulb, the central involvement of the superior olfactory pathways can only be hypothesized. 

Headache is a common symptom of viral infections, including rhinovirus, flu, and COVID-19 symptoms. Individuals may experience headache due to reduced oxygen (hypoxic headache), but longstanding headache of >6 months accompanied by olfactory dysfunction favors a neuroinflammatory mechanism. If the neuroinflammation arising from SARS-CoV-2 infection mediates headache, cognitive, and olfactory dysfunction in Long-COVID, anti-neuroinflammatory therapies may hold promise for alleviating neurocognitive symptoms and promoting olfactory recovery [38].

Combined mental clouding and headache was strongly linked to increased severity of smell alterations (OR: 41), whereas patients with mental clouding alone had an OR of 19, and those with headache had an OR of 21. SARS-CoV-2-induced inflammation of the brain [23] may contribute to various Long-COVID symptoms, including vestibulocochlear impairment [1] and persistent cough [25]. Cognitive impairment and headache may both degrade an individual’s ability to correctly identifying the odors, as memory is necessary for odor recognition. Furthermore, headache may reduce attention and concentration [39], reducing the accuracy of odor identification, especially during a taxing olfactometry analysis.

Mental clouding might affect olfactory function through several mechanisms, particularly during the phase of identification and discrimination of the odors [40,41]; the temporary loss of memory could interfere with the identification of the perceived smell, with consequent diagnosis of anosmia due to poor scores in the Sniffin’ test [9]. On the other hand, due to anatomical connections between olfactory bulbs and frontal cortex (trough pyriform cortex), the inflammation of the frontal lobe might also affect the orbitofrontal cortex, amygdala, hypothalamus, insula, entorhinal cortex, and hippocampus, areas involved in emotion and memory [42,43]. In addition, the connection between a negative emotion and the smelled odors during the test might be responsible for the parosmia referred by the patients [12].

Regarding the loss of smell, it is also important to consider the role of the nasal neuroepithelium. Although we did not identify clear signs of inflammation through the nasal endoscopy, SARS-CoV2, which belongs to SARS virus family [44], could be responsible of the destruction of the supporting cells into the neuroepithelium [45]; the loss of these cells could both cause the death of the olfactory neurons (ONs) and negatively affect their natural regeneration ability. In fact, ONs generally have extraordinary capacity to regenerate owing to robust proliferative potential of resident progenitor stem cells. The resident cells, in the adult life are retained in the olfactory bulb (and hippocampus) [43]; in patients with SARS-CoV-2, the olfactory bulbs might suffer from neuro-inflammation [46], which could both destroy the structure and reduce the capacity of the stem cells. 

We speculate that the olfactory loss might be caused by a coexistence of peripheral and central damage; SARS-CoV-2 appears to differ from other viruses of the upper respiratory system, which usually cause transitory smell loss (very rarely persistent).

We hypothesize that persistence of inflammation in the adult olfactory bulbs might induce a hypoplasia or atrophy, as shown by MRI studies [16], impeding recovery of smell; for example, children suffering from brain malformation (i.e., holoprosencephaly and septo-optic-pituitary dysplasia), whose olfactory bulbs are absent or hypoplastic, have impaired olfaction, despite the extreme brain plasticity at this age, and usually do not recover olfactory function.

### Limitations of the Study

This study was designed to critically examine those patients with chronic olfactory dysfunction and used a novel tool of mental clouding to do so, but these aspects of the design constitute significant limitations and highlight the need for future prospective studies with validated instruments. Among the caveats in this study are: (i) the absence of a comparator consisting of individuals with Long COVID but absence of olfactory symptoms, (ii) the use of an assessment designed specifically for the study, due to the lack of scales to measure mental clouding based on the subjective patients perception; this tool should be validated on a larger sample to minimize risk of bias, (iii) poor sensitivity of MMSE in the detection of small cognitive alterations, and (iv) use of cross-sectional design, which precludes causal inferences. Minor limitations were: (i) we only tested smell identification; although it was done to obtain homogeneity in the data collected in the three centers, the use of full olfactory test with thresholds and discrimination could have shown different results; (ii) data also relied on patient-reported outcomes, with potential bias in patient selection; (iii) relatively young population; (iv) despite the use of objective and validated tests, floor effects may have limited sensitivity; (v) the lack of consensus on criteria for Long-COVID in the scientific literature; (vi) the sample size skewed towards women, possibly related to the tendency for SARS-CoV-2 infection to cause less severe disease in women [33] and anosmia and smell alterations are more common among patients with mild COVID-19 [3,34].

## 5. Conclusions

Within a cohort of patients with Long-COVID syndrome, individuals with mental clouding, headache, or both, reported a more severe olfactory dysfunction compared to patients without these neurological complaint. Mental clouding might interfere with the capacity of the individual to identify odors, indirectly affecting olfactory function. Alternatively, SARS-CoV-2 neuroinflammation provides a common pathway that could explain the clustering of headache, mental clouding, and smell alteration. Pharmacologic treatments to reduce neuroinflammation along with rehabilitation are currently under investigation and may have an evolving role in alleviating suffering from headache, mental clouding, or olfactory dysfunction [30,31].

## Data Availability

Anonymized data are available under request to the corresponding author Di Stadio (ariannadistadio@hotmail.com).
